# Fabrication and Optimization of Additively Manufactured Hybrid Nanogenerators for Wearable Devices

**DOI:** 10.3390/nano15030159

**Published:** 2025-01-21

**Authors:** Khaled A. Eltoukhy, Mohamed Fawzy Aly, Marc Sarquella, Concepción Langreo, Mohamed Serry

**Affiliations:** 1 Department of Mechanical Engineering, American University in Cairo, New Cairo 11835, Egypt; eltoukhy@purdue.edu (K.A.E.); mfawzyaly@aucegypt.edu (M.F.A.); 2 Regner Engineering SLU, Ripollés 4-6, Aiguaviva, 17181 Girona, Spain; msarquella@regner.es (M.S.); clangreo@regner.es (C.L.)

**Keywords:** additive manufacturing, energy harvesting, nanocomposites, piezoelectric material, triboelectricity, hybrid nanogenerators, wearable devices, corona poling

## Abstract

This paper aims to fabricate a hybrid piezoelectric/triboelectric nanogenerator via fusion deposition modeling as a proof of concept in the wearable device industry. The nanogenerator structure consists of a TPU/ZnO nanocomposite and an Ecoflex layer. The nanocomposite layer is fabricated using two different weight percentages (15 wt% and 20 wt%) and poled piezoelectric sheets, generating 2.63 V to 3.46 V. Variations regarding the nanogenerator’s physical parameters were implemented to examine the effect on nanogenerator performance under different frequencies. The hybrid nanogenerator enabled energy harvesting for wearable devices. It was strapped on the side of the wrist to generate a potential difference with the motion of the wrist, creating a contact separation piezoelectric/triboelectric nanogenerator. Furthermore, a piezoelectric sheet was placed at the bottom of the wrist to harvest energy. The hybrid nanogenerator provided a maximum triboelectric response of 5.75 V and a maximum piezoelectric response of 2.85 V during wrist motion. The piezoelectric nanogenerator placed at the bottom of the wrist generated up to 4.78 V per wrist motion.

## 1. Introduction

The term ‘energy harvesting’ refers to the process of converting ambient energy, which would otherwise be wasted, for the storage or direct powering of low-power electronics, eliminating the need for batteries and reducing the bulkiness of the device, facilitating the use of microelectronics [1]. Piezoelectric materials are types of smart materials that can convert mechanical energy into electrical energy and vice versa. The conversion of mechanical forces or vibrations into electrical energy is known as the direct piezoelectric effect. The converse piezoelectric effect occurs when the stimuli are changed to an electrical input, resulting in a mechanical deformation [2]. Piezoelectric properties are observed due to the unique intrinsic non-centrosymmetric crystal structure of the material, which creates a charge imbalance and dipole formation under strain [3].

Piezoelectric nanogenerators (PENGs) are divided into three main types: ceramics, polymers, and nanocomposites. Piezoelectric ceramics, such as lead zirconate titanate (PZT) and zinc oxide (ZnO), are the most widely used piezoelectric materials because of their high piezoelectric coefficient. However, the main limitations of piezoelectric ceramics are their high stiffness, density, and brittleness, making them less suitable for high-strain and high-frequency applications. Polymeric PENGs are mainly used for their superior ductility and ease of fabrication, making them an ideal candidate for high-strain energy harvesting applications. Unfortunately, they exhibit lower piezoelectric coefficients compared to ceramics and single crystals, resulting in lower energy conversion efficiency and voltage output [4,5]. Lastly, nanocomposites combine nanofillers with high piezoelectric coefficients in a polymeric matrix. This approach improves power generation while maintaining flexibility and ease of polymer fabrication, thus addressing the limitations of ceramics [6].

The challenge regarding nanocomposites is in the fabrication process, which involves embedding ceramic nanofillers into a polymeric matrix, as nanofiller agglomeration poses a significant challenge. Nanofillers tend to cluster because of their large surface areas, which enhances the effects of van der Waals forces and other weak attractions between the particles. This reduces the effective surface area of the filler and weakens the interaction of the matrix, resulting in a decrease in the enhanced properties of the nanocomposite, which include mechanical and piezoelectric performance [7,8]. To break apart the agglomeration and ensure a uniform dispersion, a variety of fabrication techniques are used, such as the solution-mixing method and the compound melting method. The solution-mixing method relies on sonication to break down clusters. On the other hand, the compound mixing method uses the shear stress in the polymer melt for dispersion [9].

Piezoelectricity exhibits anisotropic behavior, making the orientation of the crystals important. During fabrication, the crystals of the piezoelectric nanocomposites are randomly oriented, leading to weak net polarization [10]. To enhance the piezoelectric response, an electric field is applied while the material is heated to enable the dipole moments to align with the direction of the electric field [2]. However, the temperature must remain below the Curie temperature of the material, above which the crystal structure is transitioned, causing loss of the piezoelectric properties [11].

Triboelectric materials are another class of energy conversion materials. Similar to piezoelectricity, triboelectricity converts energy from mechanical energy to electrical energy. While piezoelectricity is an intrinsic property, triboelectricity is a surface-dependent property that is dependent on the electrons being transferred between two contacting surfaces with different electron affinities, creating a charge imbalance that can be collected through electrodes. The generated triboelectric voltage depends on the difference in the electron affinity, the contact area, the speed, and the force of contact [12,13,14].

The primary modes of operation in triboelectric nanogenerators (TENGs) include contact separation and linear sliding. These modes differ according to the layer contact mechanism [15]. The contact separation mode (CS) generates charge through vertical motion, while the linear sliding mode (LS) relies on horizontal motion between layers [16].

Recently, hybrid piezoelectric/triboelectric nanogenerators (PTENGs) have been developed to take advantage of the similar energy conversion stimuli of both phenomena [17]. Piezoelectric materials have intrinsic properties that make them a good option in a variety of environments. However, they produce lower power compared to triboelectricity, which is capable of higher power generation but is more sensitive to environmental changes [18,19]. Integrating these materials in a hybrid nanogenerator addresses their respective limitations while ensuring energy harvesting capabilities under varying conditions [20].

Nguyen et al. conducted one of the first approaches to fabricating PTENGs using a shared electrode system to harvest energy from both the PENG and TENG layers, ensuring simultaneous strain and energy conversion [21]. This approach did not include the fabrication of a nanocomposite and instead focused on designing a system that can activate both the triboelectric and piezoelectric components in the same system. The reported peak-to-peak voltage performance of the PTENG for this approach was approximately 2.2 V. Similarly, Wang et al. created an origami-like energy generator using PVDF as a piezoelectric material and one triboelectric layer, with polypropylene film as the second layer [22]. The PVDF was polarized to increase its piezoelectric properties, and this simple change in materials resulted in an increase in the performance of the PTENG to a maximum voltage of 150 V in a simple head-to-head assembly, where the area of the device was 10 cm × 3.5 cm.

Further research focused on the use of the piezoelectric layer as one of the acting triboelectric layers in PTENGs via nanocomposite fabrication. For example, Qian et al. fabricated a nanocomposite consisting of ZnO nanoflakes and graphene in a PDMS matrix for the piezoelectric response. The second layer required for the triboelectric response was composed of Ni foam [23]. The reported peak-to-peak voltage reached a total of 122 V for the manufactured device with a size of 1 cm × 1 cm. This highlights the importance of nanocomposite fabrication in the performance of PTENGs in comparison to non-composite approaches because similar voltage magnitudes can be produced using smaller devices, which are ideal for wearable electronics. A variety of different materials can be used for the fabrication of nanogenerators, from more well-established piezoelectric nanofillers such as PZT and ZnO to novel materials such as MXenes. Kumar et al. fabricated a TiO_2_/P(VDF-TrFE)/PDMS nanogenerator [24]. The authors reported a total peak-to-peak voltage of 52 V from a 3 cm^2^ device, while Zhang et al. fabricated PTFE-PVDF/MXene nanogenerators that achieved a total voltage of 200 V [25].

One of the most underutilized methods to manufacture nanocomposites is 3D printing. Indeed, 3D printing offers flexibility in manufacturing complex shapes. Fusion deposition modeling (FDM) is one of the cheapest and simplest methods to 3D print piezoelectric nanogenerators, but the complexities involved in manufacturing a filament that is robust enough for printing is still a challenge hindering more widespread use of FDM for nanogenerator fabrication [26]. This paper aims to demonstrate a proof-of-concept hybrid piezoelectric/triboelectric nanogenerator (PTENG) fabricated using FDM for wearable devices. This method enables the fabrication of custom-sized and -shaped nanogenerator layers without the need for molding techniques or electrospinning. The prototype includes a 3D-printed ZnO/TPU hybrid layer and a casted Ecoflex-10 triboelectric sheet, designed to harvest energy from wrist motion during fist movements. Using the flexibility and speed of 3D printing, the separate triboelectric and piezoelectric performances of the PTENG were studied while varying the layer surface area, thickness, and frequency of operation.

## 2. Materials and Methods

### 2.1. Materials

TPU used eTPU-95A purchased in the form of a spool from eSun Ltd. (Shenzhen eSUN Industrial Co., Ltd., Shenzhen, China). The TPU material is used as a matrix for the ZnO nanofillers. ZnO was provided by Nano-Gate chemicals (Cairo, Egypt) with the following characteristics: a purity level of 99.9% and spherical nanofillers with an average diameter of 10 nm. The secondary layer will be created using casted Ecoflex-10 (Smooth-On ECO-FLEX 00-10) (Smooth-On, Inc., Easton, PA, USA). Tetrahydrofuran (THF) provided by Sigma Aldrich (St. Louis, MO, USA) with 99.9% purity was used as the TPU solvent.

### 2.2. Methodology

#### 2.2.1. Overview

This section provides an overview of the fabrication characterization and the supplementary testing performed to fabricate the hybrid nanogenerator. The solution-mixing method is used to fabricate the ZnO/TPU nanocomposite. TPU was dried in an oven and then dissolved in THF, and a probe sonicator was used to ensure a homogeneous dispersion of ZnO in the THF solution. The nanocomposite was then pelletized and heated to remove excess THF that could affect the quality of the filament by being trapped.

The pellets were then extruded using an extrusion machine with a die diameter of 1.75 mm, and the filament was used to print small sheets with the size of 25 mm × 15 mm × 0.5 mm to study the chemical composition via scanning electron microscope (SEM) and Fourier Transfer Infrared Spectroscopy (FT-IR). The sheets were also used to study the piezoelectric response (force voltage characterization) of the nanocomposite when loaded using known weights. Before force–voltage characterization, the specimens are poled via an electric corona poling machine to reorient the crystals in a more favorable direction for the application. A poling machine was manufactured and assembled locally, and the potential difference was applied perpendicularly to the larger face of the sheets. X-ray diffraction (XRD) is performed before and after the poling process to study the changes that may have occurred from the electric poling.

Two more sheets are printed with different surface areas (20 cm^2^ and 25 cm^2^) to be used in the hybrid layer in the nanogenerator structure, with the Ecoflex layer acting as the second layer. The two hybrid sheets are electrically poled and attached to the bed of the testing rig with the Ecoflex layer attached to the hammer. Energy collection is completed through a triple-electron setup. The setup is set to apply cyclic loading on the material at two frequencies (2 and 4 Hz). Through testing, the thickness of the Ecoflex layer is varied from 1 to 2 cm to study the effect of changing the thickness of the layer on the performance of the nanogenerator.

**Figure 1:**
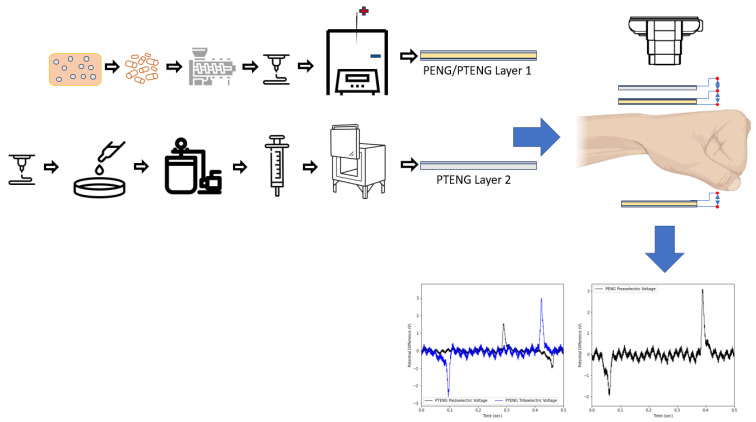
Graphical abstract of the fabrication of wearable device.

Finally, following the study on the performance of the PTENG, the hybrid nanogenerator is fabricated with a size small enough to fit comfortably on a strap, and the open-circuit voltage is measured. Both PENGs and PTENGs are strapped in different locations to be able to extract the maximum amount of energy; Figure 1 shows an abstract of the experimental process adopted in this paper.

#### 2.2.2. Nanocomposite Fabrication

**Figure 2:**
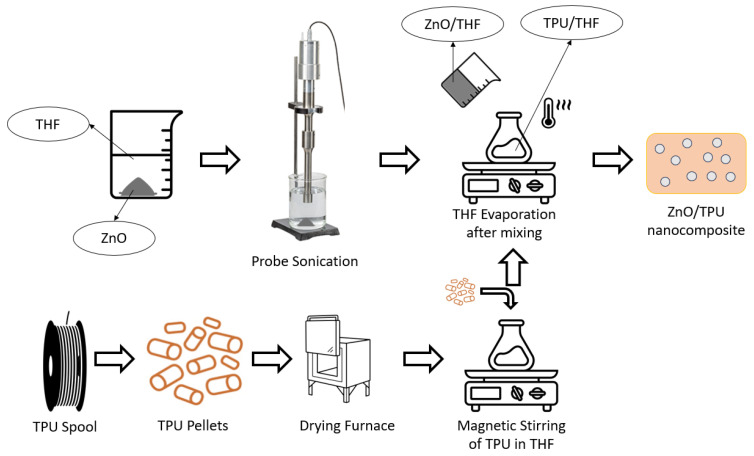
Schematic of the ZnO/TPU fabrication process.

Figure 2 shows the steps adopted to fabricate the nanocomposite. The solution-mixing method was adopted to integrate the nanofiller into the matrix with the help of a sonication process to ensure a well-dispersed homogeneous piezoelectric nanocomposite. The first step was to pelletize the TPU filament into smaller pieces to increase the surface area of the material and accelerate the dissolution of the TPU. Before dissolving the TPU in THF, it was placed in a furnace for 1 h at 100 °C to dry the pellets. The TPU was dissolved using THF in a ratio of 1:50 (liters:grams) between the THF and the TPU, respectively. The beaker containing the solution and the pellets is then placed on the magnetic stirring hotplate at 500 rpm and 40 °C and left for 1 h to ensure that all the TPU pellets have dissolved.

While the TPU is being dissolved, ZnO is dispersed in a THF solution with the help of a probe sonicator. ZnO is added to the THF solution in a ratio of 1:20 (liter:grams). The solution was placed in an ice bath to avoid evaporation of THF during sonication. The probe sonicator used was a Branson SFX550 probe sonicator (Branson Ultrasonics, Danbury, CO, USA). The sonication mode was set to the pulsated mode with a 1:1 ratio between the time on and time off when using 60% amplitude. After sonication and TPU dissolution, the two solutions are mixed and placed on the hot plate magnetic stirrer. The heater is set at 80 °C to evaporate all THF in the solution, leaving behind the TPU/ZnO nanocomposite. After the nanocomposite was extracted from the beaker, the nanocomposite block was left in distilled water overnight to wash away any THF on the surface of the composite. The block is then cut into small pellets and heated at 100 °C, while the weight is measured every 30 min. Once minimal variation in the weight is noticed, this signals that all the THF has evaporated and the material is ready to be extruded. This process is repeated using the same parameters for the 15 wt% and 20 wt% ZnO TPU composites.

#### 2.2.3. Device Fabrication

The material is fed into an extrusion machine with a mold diameter of 1.75 mm. Two 15 wt% and 20 wt% ZnO filaments were manufactured using the discussed method, and the printing parameters of both filaments were similar and consistent for all sheets printed.

The main structure of the nanogenerator consists of two layers, the ZnO/TPU 3D-printed nanocomposite layer and an Ecoflex 10 layer. The two sheets were manufactured with two different surface areas, 20 cm^2^ and 25 cm^2^, to study how the performance of the nanogenerator varies with the change in surface area. The sheets were corona-poled under a 6.5 kV potential difference using a needle diameter of 1 cm with an offset of 2 cm from the top layer of the sheet. The poling process was conducted at 150 °C for 45 min.

**Figure 3:**
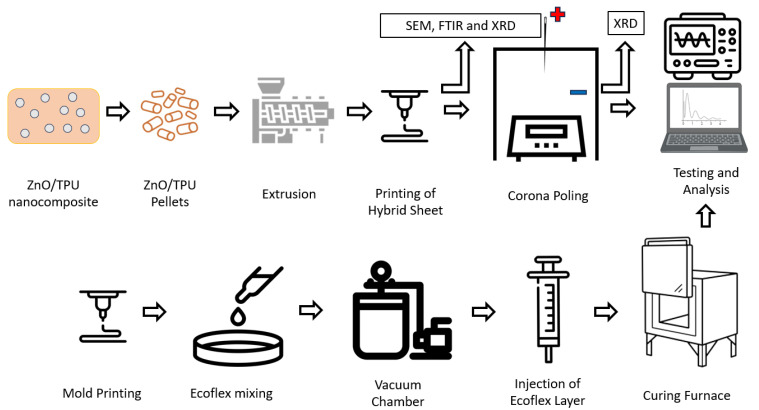
Schematic of the nanogenerator fabrication process and characterization.

Ecoflex 10 layer was injected into a closed 3D-printed PLA mold. The two components of Ecoflex were mixed using a 1:1 ratio for 5 min. The mixture is placed in a vacuum chamber before being injected into the mold and placed in an oven at 75 °C for 45 min. The two sheets are then placed on a testing rig to induce contact between the two sheets. The strain felt by the piezoelectric sheet will result in a piezoelectric response, and the contact separation mode will generate a triboelectric response. Different Ecoflex 10 layer thicknesses are fabricated to study how the thickness will affect the performance of the nanogenerators. Figure 3 shows the schematic of the nanogenerator fabrication process.

#### 2.2.4. Characterization

SEM images of the cross-sections of filaments were taken to study the dispersion of ZnO inside the filament. Neoscope (JCM-6000 Plus) JEOL benchtop SEM (JEOL Ltd., Akishima, Japan) was used to take images of pure TPU and TPU/ZnO nanocomposites. Furthermore, small sheets of 25 mm × 15 mm × 0.5 mm were printed for the characterization of both nanocomposites using Fourier Transfer Infrared Spectroscopy (FT-IR). After the characterization of the sheets, the sheets were corona-poled under the same conditions as the hybrid layer in the nanogenerator structures, and XRD was conducted before and after the poling process to determine how the poling process affected the nanocomposite. XRD was performed using a Cu anode with a K_α_ wavelength of 1.54 Å. Lastly, the small sheets were also loaded using a weight varying from 1 to 5 N to compare the voltage output between the 15 wt% and 20% nanocomposites. Pure TPU and paper sheets were used as a reference. The voltages were collected using a Tektronix MDO3204 oscilloscope (Tektronix, Beaverton, OR, USA).

## 3. Results and Discussion

### 3.1. FT-IR Analysis

**Figure 4:**
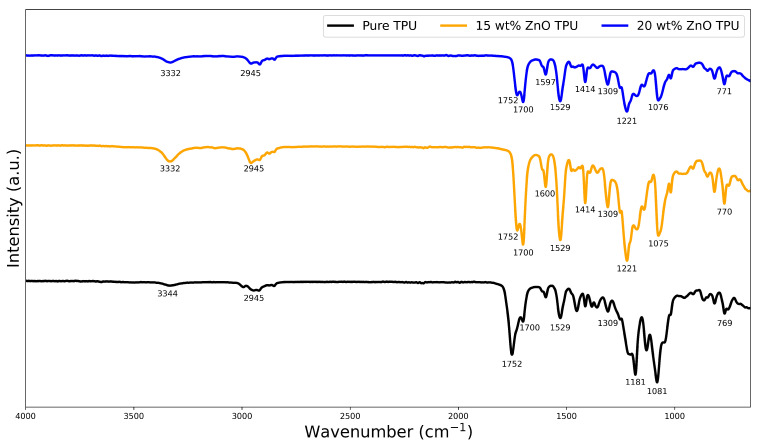
FT-IR spectra of pure TPU, 15% ZnO/TPU, and 20% ZnO/TPU.

Figure 4 shows the normalized FT-IR spectrum of the TPU compared to the nanocomposites used. In the pure TPU spectrum, broad and weak absorption peaks are observed as a result of the N–H stretching vibrations around 3336 cm^−1^. Another similar peak appeared at 2957 cm ^−1^, corresponding to the stretching vibrations of the ${CH}_{2}$ groups in the TPU structure. Strong and sharp peaks are present, recorded in the range of 1730 to 1700 cm^−1^, as a result of the stretching of C=O. Polyurethanes have a characteristic peak due to C–N stretching. This peak can be observed at 1529 cm^−1^. The peaks between 1455 and 1309 cm^−1^ are much weaker in intensity compared to the previous peaks and correspond to the deformation vibrations of the $CH_{2}$ groups present in the TPU. The stretching vibrations of the urethane group are recorded around 1118 cm^−1^. In turn, the stretching vibration of the hydrogen-bonded urethane group is presented at 1081 cm^−1^. Finally, the peaks shown between 850 and 770 cm^−1^ are associated with out-of-plane bonding vibrations of C–H bending [27,28].

The FTIR spectra of pure TPU compared to the nanocomposite showed a few key differences. The first difference was the slight shifts in the peak locations from their original positions. Furthermore, one of the main differences is the presence of more resounding peaks at approximately 1075, 1309, 1414, and 1600 cm^−1^. The 1600 cm^−1^ band could be attributed to the presence of water molecules on the exterior surface of the ZnO nanoparticles as it passed in the atmosphere, which meant that it was in direct exposure to water in the atmosphere. The band at 1438 cm^−1^ could be attributed to hydrogen-based deficiencies on the ZnO surface [29]. Furthermore, some studies expect peaks at 1090 cm^−1^, corresponding to the metal oxide stretching vibrations of Zn–O bonds [30]. The differences in the intensity of the peak can then be attributed to the presence of the ZnO bond within the matrix.

Increasing the weight percentage of the ZnO in the nanocomposite results in different peak intensities. This is evident from the different intensities between pure TPU and the nanocomposite curves, shown by the transmittance percentage of the peaks presented. The sharper peaks presented in the nanocomposite are due to the interaction between the functional group C=O and the metal atom in the nanofiller, resulting in a sharper peak at 1700 cm^−1^. Furthermore, increasing the weight percentage of the ZnO within the structure would result in the peak at 1700 cm^−1^ becoming weaker [31]. This could be attributed to the increased weight percentage pinning the chain of the TPU and preventing it from vibrating at its normal intensity. However, 1081 cm^−1^ was even more intense with the increase in ZnO% wt, which is consistent with this peak that corresponds to a Zn-O bond peak.

### 3.2. SEM Analysis

**Figure 5:**
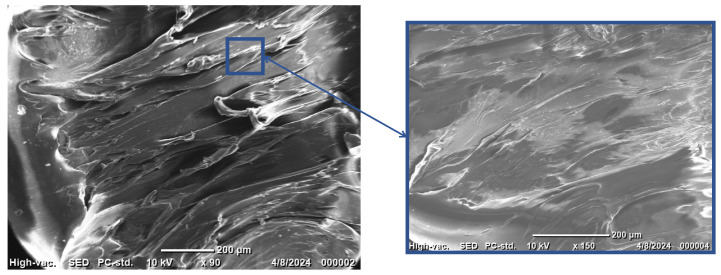
SEM image of pure TPU.

SEM images of pure TPU and the ZnO/TPU nanocomposite were taken before the extrusion process to study the degree of homogeneity of ZnO inside the TPU matrix. Figure 5 shows the SEM images of the fracture surface of the TPU provided by eSun to be used as a reference. The fracture surface appears to have experienced large deformation, which is indicated by a relatively rough surface with a non-uniform height. No inclusions could be observed as the images were taken before any addition of nanoparticles.

**Figure 6:**
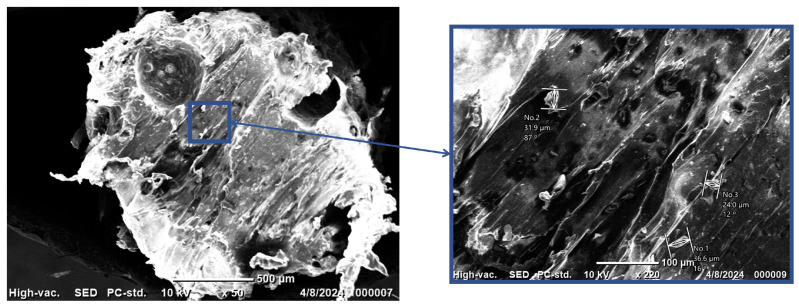
SEM image of 15 wt% Zno/TPU nanocomposite.

Figure 6 shows the SEM images of the 15 wt% ZnO/TPU nanocomposite, which shows a rougher fracture surface with a lower deformation compared to pure TPU. This indicates that the material has lost some of its ductility because of the presence of ZnO inside the matrix. Furthermore, in the nanocomposite, small inclusions could be observed spreading around the nanocomposite, indicating the presence of ZnO within the TPU matrix. The ZnO nanoparticles agglomerated, forming inclusions with an average of 30 µm. These nanoparticles are expected to enhance the piezoelectric performance of the energy harvester. The nanoparticles were evenly disturbed across the cross-sectional area, indicating a homogeneous distribution from the sonication process. Furthermore, there was no separation between the nanoparticles and the matrix at the interface, indicating good coupling between the ZnO and the TPU matrix.

**Figure 7:**
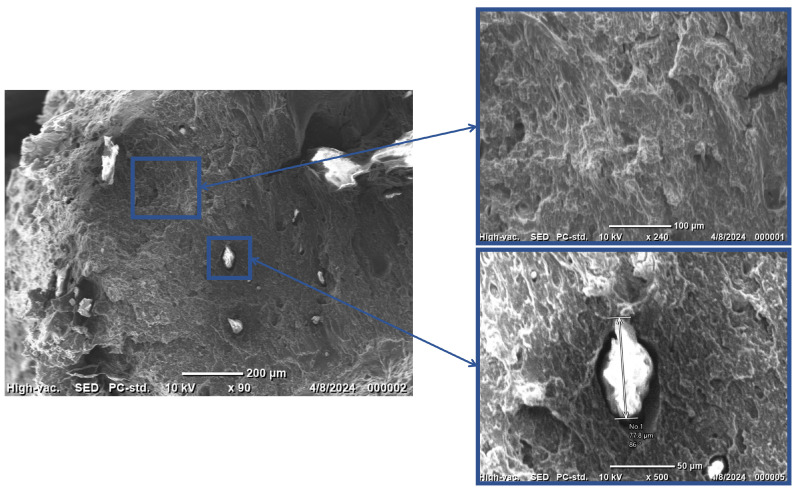
SEM image of 20 wt% Zno/TPU nanocompsite.

Figure 7 shows the SEM images of the 20 wt% ZnO/TPU nanocomposite. The images show that, due to the increased weight percentage, the size of the inclusions introduced within the matrix increased to 77.8 µm. The increased weight percentages result in more agglomeration as the attraction between the ZnO nanoparticles increases because of the increase in the surface area present. Furthermore, it is important to note that there is a gap between the ZnO phase and the matrix, which results in lower coupling and is considered to be a defect in the nanocomposite. The size of the phase might also cause an issue as, the larger and less dispersed the particles are, the more likely it is for the composite to eventually clog the nozzle of the 3D printer. Furthermore, the fracture surface showed minimal deformation compared to the 15 wt% composite and pure TPU. The loss of flexibility is due to the increased wt% of the ZnO present within the matrix.

### 3.3. XRD Analysis

**Figure 8:**
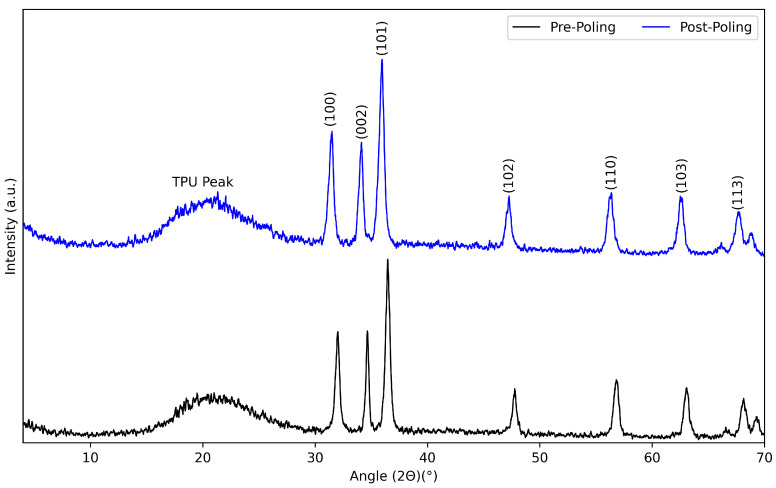
XRD patterns pre-corona and post-corona treatment.

Figure 8 shows the diffraction patterns before and after corona treatment. Both the characteristic peaks of TPU and ZnO are present in the patterns of the composite pre-corona treatment. The characteristic TPU peak was around 21°, with a minimal shift to 22° after poling, which could be due to minor reorientations or stress. This peak is broad and weak due to the lack of crystallinity of the TPU material compared to the ZnO structure peaks at 32°, 34°, and 36° for the (100), (002), and (101) planes, respectively [32,33]. Furthermore, the (100) and (101) planes increased in intensity, but the (002) plane did not show any noticeable changes.

### 3.4. Force–Voltage Characterization

To test the piezoelectric performance of the hybrid layer, incremental forces were applied to stain four different sheets monitored by a sensor. The four sheets were of the same dimensions but different materials or wt% in the case of the nanocomposites. These materials were the two nanocomposites of 15 wt% and 20 wt% to study the effect of the increased weight percentage on the piezoelectric response. The other two sheets were pure 3D-printed TPU sheets and a paper sheet to be used as references to ensure that the differences in voltages noted were due to the loading of the specimens and not to noise that is picked up by the passive probes during the test.

The loading of these sheets was performed using forces ranging from 1 N to 5 N that were measured and recorded using calibrated Arduino-powered force-sensing resistors. The sensor would continually monitor the force applied and output the change in force felt by the sensor in terms of newtons. The sensor was calibrated using known weights by applying or loading the weight on top of the sensor and changing the calibration equation until all the weights applied resulted in the correct force calculations. Each sheet was loaded five times and the average open-circuit voltage was measured.

**Figure 9:**
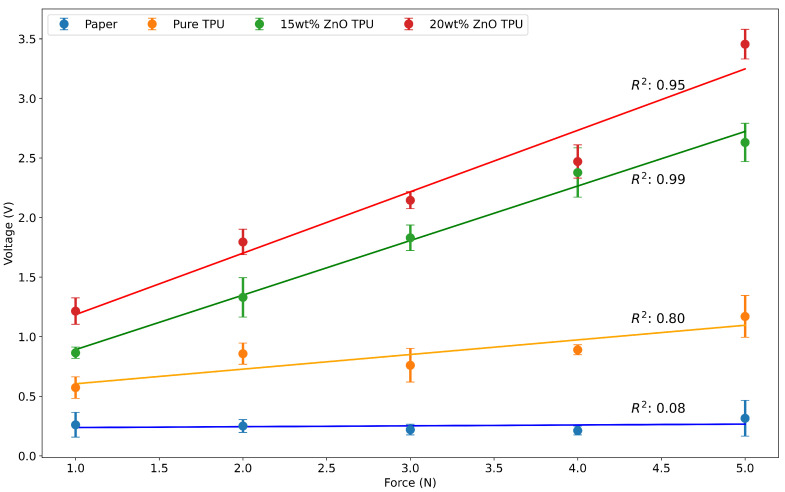
Force voltage characterization of 3D-printed piezoelectric test sheets.

Figure 9 shows the average open-circuit voltage produced when loading the sheets with the respective forces. It is noted that both the paper and the TPU show little to no piezoelectric response when loaded as there is a minimal increase in the voltage read by the oscilloscope when increasing the force on the sheet while connected. Furthermore, the recorded voltage appears to be static noise that does not vary while loading the sheet since it is in the range of microvolts and remains constant when testing the two sheets.

However, both nanocomposites show increases in the voltage measured when the material is loaded. The loading is almost linear, reaching maximum averages of 2.63 V and 3.46 V for the 15 wt% ZnO/TPU and 20 wt% ZnO/TPU nanocomposites, respectively. The greatest difference between the two compounds is observed at the highest force, where a difference of 0.825 V is recorded. However, the smallest difference is recorded at 4 N, where the difference is recorded as 0.1 V. It is important to note that none of the 15 wt% open-circuit voltages exceeded the value of the 20 wt% voltages at similar forces. This is mainly due to the wt% difference regarding the employed ZnO. Although the average was higher for the 20 wt% readings compared to 15 wt%, there was an overlap in the output voltage in the 4 N load between the two nanocomposites.

**Figure 10:**
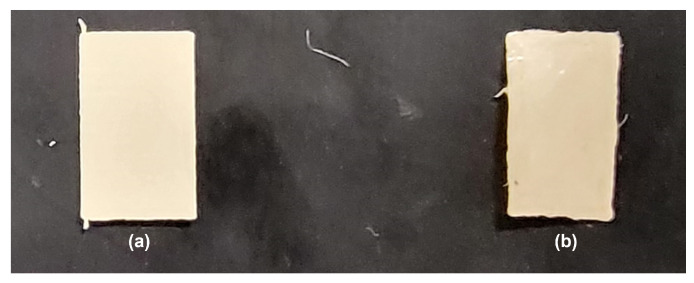
3D-printed sheets used for PENG characterization: (**a**) 15 wt% Zno/TPU; (**b**) 20 wt% ZnO/TPU sheet.

An additional increase in wt% is expected to result in more piezoelectric force. However, because of the constant clogging of the nozzle, this could not be tested properly. Multiple failures occurred during the printing of the 20 wt% nanocomposite filament due to the agglomeration of the material and the stiffness of the filament causing either nozzle clogging or filament fracture due to brittleness at the extruder. As a result, 15 wt% was chosen for the hybrid nanogenerator application even though 20 wt% is expected to produce more energy. An image of the two sheets used in testing is shown in Figure 10, demonstrating the morphology of the sheets. Evidently, increasing the loading of the ZnO would result in a more deformed structure. A change in morphology along with the premature failure of the filament due to the larger agglomeration, observed in Figure 7, indicate that 20 wt% ZnO is the threshold for printing ZnO within a TPU structure.

### 3.5. Nanogenerator Performance Optimization

**Figure 11:**
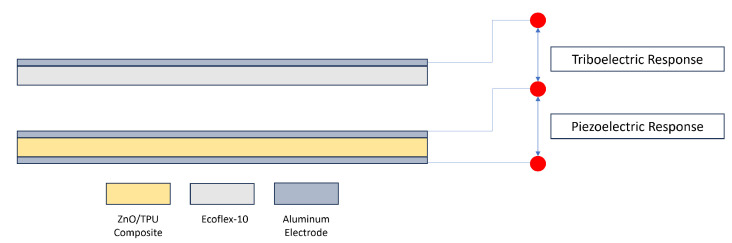
Schematic of the three-electrode system.

The hybrid sheet was placed on a bed with aluminum (Al) electrodes and copper (Cu) wires attached to the top and bottom sides of the sheet. The Ecoflex layer was then placed on the hammer of the setup with Al electrodes on the interface between the hammer and the layer. Figure 11 shows a schematic of the three-electrode system and the wire connection used to measure the piezoelectric and triboelectric responses [34].

**Figure 12:**
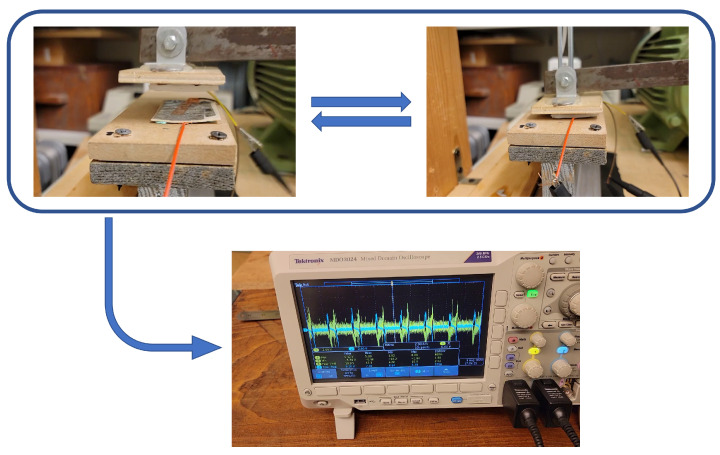
Testing of the PTENG performance.

The test was repeated using three different Ecoflex layer thicknesses (1 cm, 1.5 cm, and 2 cm). The Ecoflex layers were tested three times against the same piezoelectric sheets, and the average piezoelectric and triboelectric responses were recorded. Performance was measured using two different loading frequencies (2 Hz and 4 Hz). The surface area (to match the surface area of the hybrid layer) and sheet thickness of the Ecoflex sheet were varied to study the effect of this variation on PTENG performance. Figure 12 shows how the PTENG performance was measured during the optimization process.

**Figure 13:**
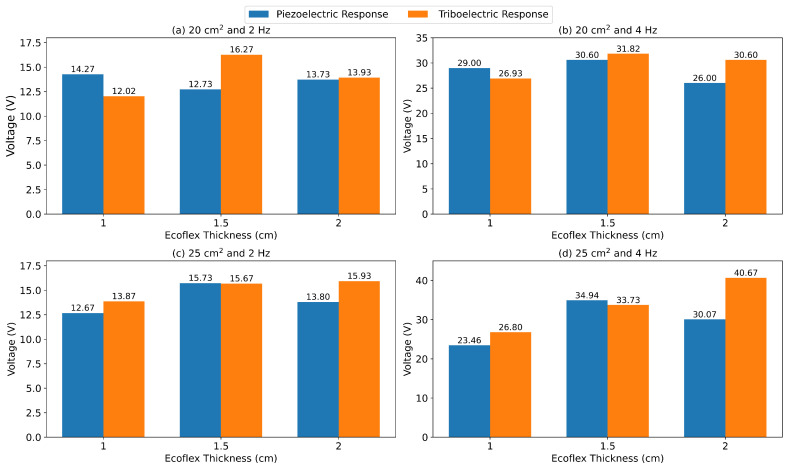
PTENG performances of the 20 cm^2^ generator under (**a**) 2 Hz and (**b**) 4 Hz loading, the 25 cm^2^ generator under 2 Hz (**a**) and 4 Hz (**b**) loading, and the 25 cm^2^ generator under (**c**) 2 Hz and (**d**) 4 Hz loading.

Figure 13 shows the performance of the 20 cm^2^ and 25 cm^2^ PTENGs under 2 and 4 Hz cyclic loading when varying the thickness of the Ecoflex sheet and the surface area of the nanogenerator. As the surface area of the nanogenerator increases, the piezoelectric and triboelectric responses are expected to increase because of the increase in nanofiller weight in the larger volume and the larger surface area, respectively. However, the piezoelectric and triboelectric effects showed minimal variations under a specific loading frequency. The minimal change in the piezoelectric response (which is a strain-dependent response) could be attributed to the larger surface area minimizing the deformation experienced by the hybrid layer at all the Ecoflex thicknesses. A similar explanation could be used for the triboelectric response as the minimal deformation would result in both an improper contact area and less deformation (growth) in the x- and y-directions according to the Poisson ratio. Although higher responses were expected, a larger surface area under a fixed force would mean smaller deformation and a decrease in the piezoelectric response and triboelectric response.

When Ecoflex thickness was varied, the triboelectric response of the nanogenerator showed that, in the lower surface areas, an optimum was reached at an Ecoflex thickness of 1.5 cm. This was observed in both the higher and lower frequencies of the nanogenerator operation. For larger surface areas, the triboelectric response continued to increase for both operating frequencies. Thus, there may be an optimum value for the triboelectric response at a larger thickness, hinting at a possible shift in the optimum thickness when varying the Ecoflex layer thickness. Similar behavior could be noticed for the piezoelectric response with the execution of the 20 cm^2^ PTENG under 2 Hz frequency loading. This could be due to the better load transfer that occurs when the thickness of the Ecoflex layer is increased. At higher thicknesses, the Ecoflex layer thickness can ensure better contact and load transferring without the excessive deformation of the elastic Ecoflex layer. At higher thicknesses, the material loses the ability to deform to a similar extent to the hybrid layer, causing a mismatch in terms of contact surface area and deformation, resulting in lower triboelectric and piezoelectric responses.

Higher frequencies would result in higher forces being applied and subsequently greater deformation. In every case, the piezoelectric and triboelectric responses increased with an increase in the operation frequency when fixing the Ecoflex thickness and surface area. Increasing the applied force is the easiest method to harvest more energy from a PTENG. Unlike the other parameters studied, there was no optimal frequency value as there is no geometrical constraint that was required to be satisfied, such as conservation of mass and Poisson ratio, when varying the geometry of the structure. More work needs to be completed to study how more extreme variations could lead to more noticeable differences and more concrete conclusions.

Triboelectric responses are expected to generate a larger potential difference than piezoelectric responses. However, during the testing, this was not the case for many of the combinations presented, and the TENG response only became more pronounced at higher Ecoflex thickness values and frequencies. Triboelectric responses are a function of the difference in electron affinity of the layers used. In this structure, TPU acts as a positively charged layer and the Ecoflex acts as a negatively charged layer [35,36]. However, Ecoflex has lower electron affinity compared to the more commonly used negatively charged materials such as PTFE and PDMS, resulting in lower overall electrification during contact and, as a result, lower energy for the TENG response [37,38]. Also, TPU has the same issue compared to metallic materials such as Al and Cu, which are considered to be the main electron-donating layers. This can be further confirmed by examining the results generated by Ecoflex-based TENGs from the literature. Zheng et al. fabricated a Cu-Ecoflex-based nanogenerator that was able to generate voltages up to 17 V [36]. Basith et al. reported a similar range of voltages when using pure Ecoflex with Al and were only able to improve the performance after modifying the Ecoflex layer for more charge generation [39].

### 3.6. PENGs and PTENGs as Wearable Energy Harvesters

**Figure 14:**
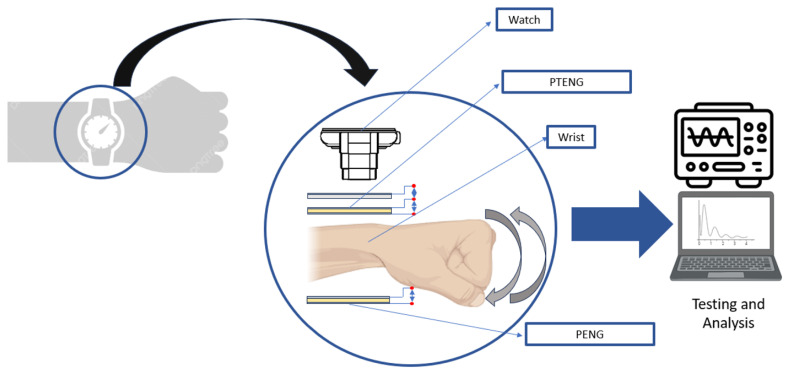
Schematic of PENG and PTENG placement during testing.

For the proof of concept, the 15 wt% ZnO/TPU nanocomposite and an Ecoflex thickness of 1.5 cm were used. The optimization process indicated that it could include the optimal combination of parameters for low frequency and lower surface areas. The Ecoflex sheet and the nanocomposite sheet have a surface area of 3.75 cm^2^ to be able to fit comfortably on the strap. Figure 14 shows the placement of the PTENG and PENG on the wrist along with the necessary connections needed to harvest the energy generated. The force sensors were placed in the same position and used to obtain a reading of the maximum force applied on the PENG and PTENG during wrist motion. The force sensors estimated that the force applied during the wrist motion on the PENG is approximately 10 N, resulting in a potential difference. For the hybrid sheet, the piezoelectric component was experiencing much lower forces that reached a maximum of 2 N.

A PTENG structure consisting of an Ecoflex sheet and 15 wt% ZnO/TPU layers was placed on the side of the wrist for energy harvesting. The motion of the wrist would activate the PTENG via compression and bending of the hybrid sheet, with a triboelectric response via a mix of contact separation and layer sliding between the two layers. A PENG was also placed at the bottom of the wrist, prestrained for improved energy harvesting over wrist motion. PTENG values were reported up to a maximum of 2.85 V, and 5.74 V was recorded for the piezoelectric and triboelectric responses. However, the PENG resulted in a maximum voltage of 4.78 V due to the extra loading applied on the sheet during straining.

The potential difference values for the PENG and PENG were monitored using a Tektronix MDO3204 oscilloscope, and the open-circuit voltages were measured. The PENG showed a maximum peak-to-peak of approximately 4.780 V when placed under the wrist to experience both compressive force and bending force, with a resultant 10 N. For the PTENG, a lower piezoelectric response of 2.85 V and a triboelectric response of 5.74 V were recorded.

**Figure 15:**
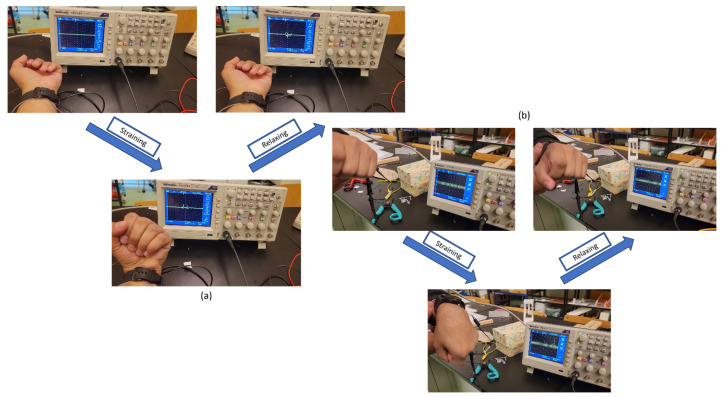
Testing of (**a**) PENG and (**b**) PTENG responses.

**Figure 16:**
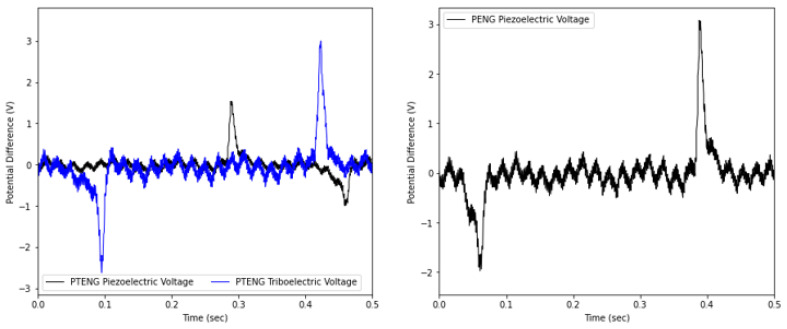
Open-circuit voltage recorded during testing.

The piezoelectric response is always reported to be observed before the triboelectric response due to compression, resulting in an instant potential difference due to the straining of the ZnO. For the triboelectric response, there is always a lag, considering that the layers are still in contact, and the charge is only generated when the layers are separated due to the charge imbalance. Figure 15 shows the testing of the fabricated PENG and PTENG connected to the oscilloscope and Figure 16 the recorded open-circuit response recorded during wrist motion.

The simultaneous use of both the PENG and PTENG would result in a better response. The PTENG was placed at the top of the wrist where there would be lower strain forces and the use of the TENG could enhance the energy output. The PENG was placed at the bottom near the strap fastener, resulting in higher loading and thus more applied strain. This is reflected by the differences in piezoelectric response in Figure 16.

## 4. Conclusions

Hybrid nanogenerators were fabricated by embedding ZnO (15 wt% and 20 wt%) within a TPU matrix. The TPU/ZnO material was then extruded into a filament for 3D printing of an efficient PENG layer after the corona poling treatment. The PENG generates around 2.63 V or 3.46 V depending on the weight percentage of ZnO used when applying 5 N. Both PENGs follow a linear increase in voltage produced based on the load applied to the material. The increase in voltage is attributed to the increase in the ZnO nanofiller within the material, producing more charges that could be collected by the electrodes after poling. All the nanocomposite layers were 3D printed, and, through multiple iterations, it became clear that the 20 wt% is not suitable for larger and more complex printing that would require a longer printing time due to constant clogging of the nozzle or breaking of the filament, causing the print to fail.

PTENG performance was optimized by applying forces under different frequencies, sheet thicknesses, and surface areas. The results show that the triboelectric performance tends to reach an optimum when the thickness of the Ecoflex sheet is varied. This could be observed at 20 cm^2^ at any loading frequency. For the 25 cm^2^ one, the optimum was yet to be reached as the response increases as the Ecoflex thickness increases, indicating that the triboelectric response optimum might shift due to surface area changes. Lastly, the triboelectric and piezoelectric responses are always higher for higher-frequency loading. For the triboelectric and piezoelectric responses, increasing the surface area demonstrated a small, almost negligible, increase in response followed by a decrease, with no apparent trends. This could be explained by the decrease in deformation experienced by each layer as the surface area decreased and a fixed force was applied.

A PTENG structure consisting of an Ecoflex sheet and 15 wt% ZnO/TPU layers was placed on the side of the wrist for energy harvesting. The motion of the wrist would strain the hybrid layer, producing a piezoelectric response, a triboelectric response, and a triboelectric response simultaneously. Another PENG was also placed at the bottom of the wrist. PTENG values were reported up to a maximum of 2.85 V, and 5.74 V was recorded for the piezoelectric and triboelectric responses. The PENG resulted in a maximum voltage of 4.78 V due to the extra load applied to the sheet during straining.

## Data Availability

Data are contained within the article.
